# Introduced plants of *Lupinus polyphyllus* are larger but flower less frequently than conspecifics from the native range: Results of the first year

**DOI:** 10.1002/ece3.6964

**Published:** 2020-10-29

**Authors:** Satu Ramula, Aino Kalske

**Affiliations:** ^1^ Department of Biology University of Turku Turku Finland

**Keywords:** climate gradients, intraspecific variation, invasive species, plant traits, rapid evolution

## Abstract

Introduced species, which establish in novel environments, provide an opportunity to explore trait evolution and how it may contribute to the distribution and spread of species. Here, we explore trait changes of the perennial herb *Lupinus polyphyllus* based on 11 native populations in the western USA and 17 introduced populations in Finland. More specifically, we investigated whether introduced populations outperformed native populations in traits measured in situ (seed mass) and under common garden conditions during their first year (plant size, flowering probability, and number of flowering shoots). We also explored whether climate of origin (temperature) influenced plant traits and quantified the degree to which trait variability was explained collectively by country and temperature as compared to other population‐level differences. Three out of four plant traits differed between the native and introduced populations; only seed mass was similar between countries, with most of its variation attributed to other sources of intraspecific variation not accounted for by country and temperature. Under common garden conditions, plants originating from introduced populations were larger than those originating from native populations. However, plants from the introduced range flowered less frequently and had fewer flowering shoots than their native‐range counterparts. Temperature of a population's origin influenced plant size in the common garden, with plant size increasing with increasing mean annual temperature in both native and introduced populations. Our results of the first year reveal genetic basis for phenotypic differences in some fitness‐related traits between the native and introduced populations of *L. polyphyllus*. However, not all of these trait differences necessarily contribute to the invasion success of the species and thus may not be adaptive, which raises a question how persistent the trait differences observed in the first year are later in individuals’ life for perennial herbs.

## Graphical abstract

We explored trait changes in the perennial herb Lupinus polyphyllus based on 11 native populations in the western USA and 17 introduced populations in Finland. Our results of the first‐year reveal genetic basis for phenotypic differences in some fitness‐related traits between the native and introduced populations of *L. polyphyllus*, but also raise a question how persistent the trait differences observed in the first year are later in individuals’ life for perennial herbs.

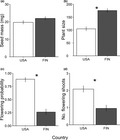

## 1 INTRODUCTION

Introduced species can be free from many of the ecological and evolutionary constraints present in their native range, such as natural enemies and competitors that are adapted to their presence (e.g., Callaway & Aschehoug, 2000). As a consequence, introduced species may provide valuable information on trait evolution and species responses to novel environments (e.g., Colautti & Lau, 2015; Parker et al., 2003). Numerous studies that have compared plant traits between native and introduced populations have reported differences (e.g., Buckley et al., 2003; Ebeling et al., 2008; Turner et al., 2014; Zhang et al., 2018), with introduced plants tending to be larger and more fecund than their conspecifics from the native range (reviewed in Parker et al., 2013). Given that these traits can affect population dynamics, such trait changes can contribute to a higher population growth rate and faster population spread in species’ introduced ranges. Understanding the causes of trait changes in introduced species is therefore essential for predicting shifts in plant communities under global environmental change.

One potential explanation of differences in plant traits observed in situ between native and introduced populations could be that they arise from rapid evolution in the introduced range in response to changes in the abiotic and biotic environment (Colautti & Lau, 2015). For example, a lack of specialist enemies (e.g., herbivores, pathogens) in new environments might enable individuals to invest more in growth and fecundity instead of defense against enemies (Blossey & Nötzold, 1995; Joshi & Vrieling, 2005). Since many invasive plant species are originally introduced as ornamentals (Hulme et al., 2018), trait differences can also result from pre‐introduction evolution due to horticultural breeding (Kitajima et al., 2006; te Beest et al., 2012). Alternatively, phenotypic differences between native and introduced populations could be environmental in origin, arising as a result of more favorable growth conditions in the introduced range (Moloney et al., 2009). As an example, in the annual *Conyza canadensis*, individuals in the introduced range outperformed those in the native range, but the two groups of plants performed similarly when they were exposed to a common environment (Rosche et al., 2019), suggesting that trait differences were phenotypic rather than genetic. As these results indicate, in order to reveal the potential genetic basis (if any) for the success of invasive populations, a comparison under standardized conditions is necessary (Moloney et al., 2009).

Plant traits are highly variable within species, with a significant proportion of the variability arising from differences among populations within both native and introduced ranges (e.g., Ebeling et al., 2008; Rosche et al., 2019); for this reason, it is necessary to sample multiple populations from each range. Climate can be the main selective force for plant traits, resulting in phenotypic variation along climatic clines. Such clinal variation is expected particularly for native populations due to their long evolutionary history. Introduced populations, in turn, may either breakdown environmental constraints (Endriss et al., 2018; Smith et al., 2020) or may adapt to local abiotic conditions rapidly, showing parallel climatic clines to their native counterparts (e.g., Hodgins & Rieseber, 2011; McGoey et al., 2020). It is therefore preferable that analyses of plant performance also consider differences in abiotic conditions that may contribute to trait variability (Colautti et al., 2009; Rosche et al., 2019).

Here, we explore trait differences in the perennial herb *Lupinus polyphyllus* (Lindl.) based on 11 native populations in the western USA and 17 introduced populations in Finland. More specifically, we investigated variation in seed mass in situ, and variation in plant size, flowering probability, and number of flowering shoots during the first growing season under common garden conditions in the introduced range. All four traits are key components of the population dynamics of the species and are thus closely related to its fitness (Ramula, 2014; Sõber & Ramula, 2013). We asked three questions: (a) Do plants from the introduced populations differ those from the native populations in terms of plant traits? (b) Does climate of origin influence plant traits similarly in both native and introduced populations? (c) How much trait variability is explained collectively by country and climate as compared to other sources of intraspecific variation? We predicted that plants from the introduced populations would be larger in size and would have higher flowering probability and flowering shoot production than those from the native population. Moreover, due to their longer evolutionary history, we predicted that plants from the native populations would exhibit a stronger association with climate of their origin than plants from the introduced populations. Finally, we predicted that all traits would exhibit population‐level variation not explained by country and climate.

## 2 MATERIALS AND METHODS

### 2.1 Study system, DNA barcoding, and climate variables

*Lupinus polyphyllus* (garden lupin, Fabaceae) is a 50–100 cm high, short‐lived perennial herb that is native to parts of western North America that have mostly an oceanic climate; it is invasive in Europe, southern Australia, New Zealand, and Chile (Fremstad, 2010; Meier et al., 2013). In Finland, the species was recorded as a garden escaper in the southern parts of the country in the late 1800s (Fremstad, 2010), and it is currently associated with declines in local flora and insect fauna (Valtonen et al., 2006; Ramula & Pihlaja, 2012; Ramula & Sorvari, 2017). It inhabits moist meadows and river banks in the native range (Beuthin, 2012), and road verges, wastelands, and forest understories in the introduced range (Fremstad, 2010). The species reproduces mostly by seed but vegetative reproduction via rhizomes is possible (Li et al., 2016). An individual plant is able to produce hundreds of seeds (Aniszewski et al., 2001; Ramula, 2014) which are dispersed ballistically up to a few meters from the mother plant (Jantunen et al., 2005) and may remain viable in the soil for decades (Fremstad, 2010).

In July–August 2018, we collected seeds from 16 putatively native populations in the western USA and 17 introduced populations in Finland across a latitudinal gradient. In the native range, unlike in Finland, several *Lupinus* species co‐exist. Because the American populations were visited only once for seed collection (outside the flowering season), we used a standard molecular barcoding of leaf samples collected from the seedlings in the greenhouse (see below) to confirm species identity. Species identity for the Finnish populations was determined in the field during the flowering period. DNA of 3–4 individuals per population (66 samples in total) was extracted from frozen leaf samples using NucleoSpin Plant II‐kit (Macherey‐Nagel) following the manufacturer's instructions. DNA barcoding was done by amplifying a short fragment of the ITS region using primers ITS2_S2F and ITS2_S3R (Chen et al., 2010). The PCR consisted of 1X QMP Master Mix (Qiagen), forward and reverse primer each at a final concentration of 0.2 μM, 1 μl template DNA and PCR grade water, in a total reaction volume of 12 μL. The amplification profile included an initial denaturation at 95°C for 15 min., followed by 35 cycles of denaturation at 98°C for 60 s, annealing at 58°C for 90 s, and extension at 72°C for 60 s. Prior to sequencing, the PCR products were enzymatically purified with A’SAP PCR clean up kit following the manufacturer's protocol (ArcticZymes). The purified samples were sent to Macrogen Europe for Sanger sequencing.

We retrieved 310 ITS1 sequences from Genbank for 117 available *Lupinus* species, subspecies or varieties. These sequences were aligned and trimmed in Geneious 2019.2.3 to build an UPGMA tree from pairwise distances based on the HKY substitution model. This tree, together with the phylogeny established by Eastwood et al. (2008), was used to restrict the dataset to 159 samples from 56 more closely related species, subspecies or varieties. To establish the relationships between individuals from these 56 species and our samples, we built a sequence tree in Beast v. 2.4.8 (Bouckaert et al., 2019), based on the birth‐only (Yule) model—this choice being motivated by the relatively shallow evolutionary history of the *Lupinus* genus. This model was parametrized with the HKY substitution model (with 4 gamma‐distributed rate classes and a non‐null proportion of invariant sites), and a strict molecular clock (due to a small number of informative sites in our sequence data). The MCMC algorithm was run for 100,000,000 generations, sampling every 1,000th state, which was largely sufficient to reach convergence. Resulting trees were summarized through maximum clade credibility, locating nodes at their common ancestor height in the posterior distribution. For visualization, we generated a minimum‐spanning network in popArt (Leigh & Bryant, 2015) by grouping haplotypes based on different assignment criteria in the Yule tree topology (see Dryad Digital Repository https://doi.org/10.5061/dryad.rjdfn2z8f for details).

DNA barcoding revealed that assignment to *L. polyphyllus* was unclear for five American populations (i.e., ITS1 alone did not allow us to conclude on species identity in our dataset); these populations were therefore omitted from analyses and we were left with 11 populations from the native range (Table 1). The range of between‐population distances was 3–1061 km (mean = 441 km) in the USA and 1–441 km (mean = 213 km) in Finland. The native populations generally inhabited lower latitudes and higher altitudes than the introduced populations (Table 1).

**Table 1:** Seed sampling locations of the perennial *Lupinus polyphyllus* in two countries, representing native (USA) and introduced (FIN) populations. No. plants/No. mothers denote sample sizes in the common garden experiment and in the seed mass analysis, respectively. Note that No. plant is missing in some populations due to poor germination

Pop	Country/region	Lat, Long	Altitude (m)	Mean annual temp (C°)	No. plants/ No. mothers
1	USA/Utah	37.85, −109.47	3,143	3.42	‐/20
2	USA/ California	38.18, −120.04	1574	9.10	20/20
3	USA/ California	38.32, −119.66	2,806	1.99	9/9
4	USA/California	38.32, −119.69	2,419	3.55	6/13
5	USA/Utah	38.41, −109.22	2,801	4.52	‐/15
6	USA/ California	38.69, −120.02	2,467	3.83	17/20
7	USA/ California	39.35, −120.35	2,235	4.68	14/18
8	USA/ California	39.43, −120.24	1947	5.80	13/20
9	USA/ California	39.71, −120.99	1562	8.65	20/20
10	USA/ California	41.17, −120.15	2,375	3.85	‐/20
11	USA/Oregon	42.46, −122.40	1,093	8.95	20/20
12	FIN/Turku	60.36, 22.27	33	5.42	12/20
13	FIN/Turku	60.41, 22.74	38	5.06	14/20
14	FIN/Turku	60.43, 22.39	44	5.08	14/20
15	FIN/Turku	60.48, 22.19	18	5.23	12/20
16	FIN/Turku	60.48, 22.20	20	5.23	15/20
17	FIN/Turku	60.51, 22.29	43	5.05	‐/20
18	FIN/Turku	60.52, 22.35	28	5.12	15/20
19	FIN/Lahdesjärvi	61.46, 23.78	127	4.08	15/20
20	FIN/Jämsä	61.85, 25.17	92	3.66	20/20
21	FIN/Vaajakoski	62.24, 25.89	97	3.32	17/20
22	FIN/Hankasalmi	62.28, 26.34	112	3.08	15/20
23	FIN/Kuopio	62.62, 27.12	104	2.83	17/20
24	FIN/Kuopio	62.66, 27.34	120	2.77	13/20
25	FIN/Kuopio	63.13, 27.99	168	2.42	17/20
26	FIN/Kuopio	63.28, 27.68	140	2.41	17/20
27	FIN/Kuopio	63.31, 27.45	100	2.42	15/20
28	FIN/Kuopio	63.36, 27.75	136	2.42	17/20

To explore whether climate of origin influences plant traits, we obtained data on mean annual temperature, mean temperatures of warmest and coldest quarters, and mean annual precipitation for each population from WorldClim version 2 (Fick & Hijmans, 2017) using the package raster (Hijmans, 2019) in R software (R3.5.3; R Development Core Team, 2019). All these climate variables were based on average monthly climate data from 1970 to 2000 with a spatial resolution of about 1 km^2^. We chose mean annual temperature to present temperature as it correlated with temperatures of warmest and coldest quarters (*r* = 0.85 and *r* = 0.97, respectively). Mean annual temperature varied across the study populations (Table 1), but did not differ between native and introduced ranges (*t* = −1.79, *p* = .096, *t* test; 5.30°C ± 2.38 versus. 3.87°C ± 1.90, respectively). Precipitation was not included in the analysis due to a small overlap between countries; mean annual precipitation was higher in the native populations than in the introduced populations (*t* = −2.41, *p* = .031, *t* test; 70.16 mm ± 20.75 (*SD*) versus. 54.63 mm ± 3.41, respectively) and this climate variable was thus confounded with country. Moreover, mean annual precipitation correlated positively with mean annual temperature (*r* = 0.68).

### 2.2 Trait variability

After air‐drying the seeds for about two months at room temperature, we individually weighed 10 fully developed, randomly chosen seeds per mother plant (if possible) to quantify seed mass and stored them in paper bags for later use. Flat, wrinkled seeds were not considered. In mid‐January 2019, we chose two seeds from each mother plant for a growing experiment at the Ruissalo Botanical Garden of the University of Turku (lat, long = 60.43, 22.18). To promote germination, we scarified each seed by nicking the seed coat with a scalpel (Beuthin, 2012). The scarified seeds were sown individually into plastic trays (16 × 16 pots of 2 × 2 cm) filled with a commercial potting mix suitable for seedlings (Kekkilän taimimulta). The trays were kept in a greenhouse at 15°C in the daytime and 12°C at night with a photoperiod of 16 hr light and 8 hr dark and were watered when necessary. Two weeks later, seedlings that emerged were replanted into larger plastic pots of 8 × 8 cm (volume of 0.3 L; preferably one seedling per mother plant) filled with a commercial potting mix for garden plants (Kekkilän karkea ruukutusseos) and were kept in the greenhouse. Due to poor seed germination in some of the populations, we repeated seed scarification and seed sowing for two more seeds from 1 to 20 mother plants from 15 populations at the end of January 2019. This time, the scarified seeds were placed on a moist paper towel in petri dishes in the greenhouse for a week, and seedlings were then planted individually in 8 × 8 cm plastic pots.

After excluding three native populations and one introduced population due to poor germination (see Table 1 for populations), the growing experiment consisted of 8 populations from the native range and 16 populations from the introduced range (6–20 seedlings per population for a total of 364 plants). The distances between the remaining populations were 3–476 km (mean = 174 km) in the USA and 1–441 km (mean = 213 km) in Finland. Eight populations contained two seedlings from 1 to 5 mother plants, while the rest of the populations contained a single seedling per mother plant.

At the end of May, when night frosts were unlikely, the plants were replanted into plastic pots (volume of 1.2 L) and moved to a common garden. They were watered regularly during the growing season, but no fertilizer was added. In late May, and again in the beginning of August, the following plant traits were recorded: survival, plant height measured from the base to the tip of the tallest leaf (cm), diameter at the base (cm), flowering probability (a plant having a flowering shoot or shoots), and number of flowering shoots. Furthermore, this last trait was recorded every other week, at which time new shoots were removed to prevent cross‐pollination and gene flow between plants in the common garden and natural populations nearby. Consequently, the plants did not produce seeds in the common garden. Due to asynchronous flowering (the native populations tending to flower earlier than the introduced populations), flowering shoots were often removed from different rather than repeatedly from the same individuals. As the removal of flowering shoots itself may induce flowering, we used the first record from each individual for the analysis of flowering shoots because this measure reflects fecundity under natural conditions (the total number of flowering shoots during the experiment produces qualitatively similar results to this conservative measure, results not shown). In the end, plant survival was excluded from statistical analyses as only 3 out of 364 plants died during the experiment (99.2% survival).

### 2.3 Statistical analyses

To ensure that the data on four plant traits considered (seed mass, plant size, flowering probability, and number of flowering shoots) were not spatially structured, we assessed potential spatial autocorrelation in the residuals of each model (see below) based on Moran's I correlograms with 1,000 permutations (using ncf::correlog; Bjornstad & Cai, 2019 in R software). No evidence for spatial autocorrelation was detected for distance classes of 10 km (*r* < 0.30).

To examine differences in the four plant traits between the native and introduced populations, and their relationship with temperature of a population's origin, we conducted linear or generalized linear mixed models. For seed mass (logarithmically transformed), we fit a linear mixed model (using lme4::lmer; Bates et al., 2015) with the fixed explanatory variables of country (USA, Finland), mean annual temperature (a continuous variable), and the interaction between country and temperature. The model contained the random factors of population and mother plant nested within population in order to consider multiple observations from the same mother. We also fit a linear mixed model for plant size (height × base diameter in cm) measured in August, the variable was square root transformed to normalize residuals. Flowering probability (flowered or not during the entire experiment) was analyzed with a binomial logit‐link generalized linear mixed model (GLMM) and the number of flowering shoots (based on flowering plants only) was analyzed with a Poisson log‐link GLMM (lme4::glmer). Plant size in May (square root transformed) was used as a covariate in the GLMMs. In all statistical models, country was included as a fixed factor, mean annual temperature was used as a continuous fixed factor, and the interaction between country and temperature was also considered. Population was included as a random factor. Model assumptions were verified from residual plots (the linear models) and a dispersion parameter (GLMMs; the dispersion parameters were 0.53 and 0.90). Significance of the fixed factors was evaluated with a type II Wald's test (using car::ANOVA; Fox & Weisberg, 2019).

To quantify how much trait variability was explained collectively by country and temperature as compared to other sources of intraspecific variation, we calculated marginal and conditional *R* ^2^ values for each trait (using MuMIn::r.squaredGLMM; Bartón, 2019). The former describes the proportion of the total variance explained by the fixed factors (country and temperature), while the latter describes the proportion of the total variance explained by both the fixed and random (i.e., population and mother plant) factors.

## 3 RESULTS

**FIGURE 1:**
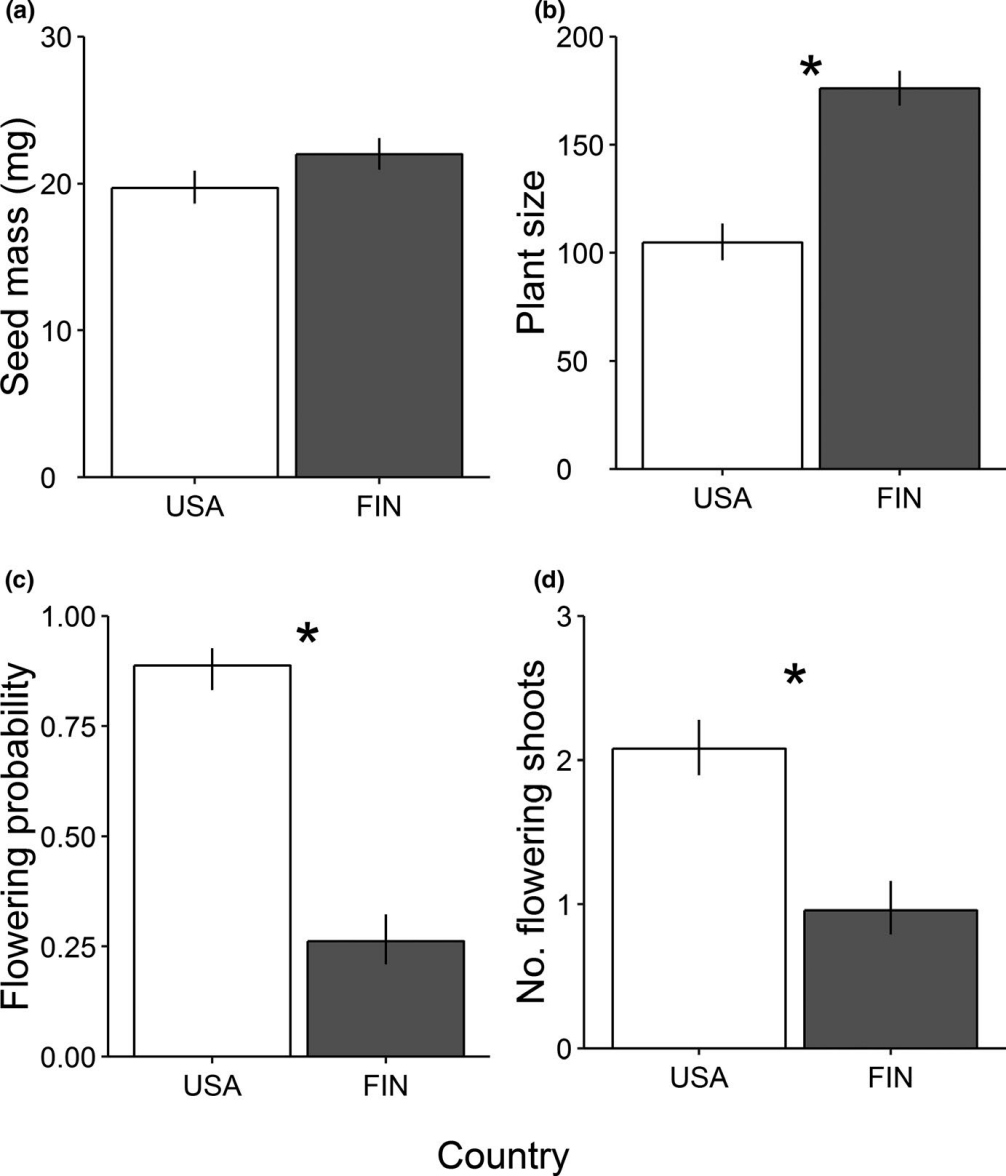
Traits of the perennial *Lupinus polyphyllus* (back‐transformed least square mean ± SE) in native (USA) and introduced (FIN) populations. Seed mass was estimated in situ, while the other traits were estimated under common garden conditions in the first summer. Plant size was measured as height × base diameter in cm. An asterisk denotes a significant difference between countries (*p‐*value < .05) based on a Wald's test

Three out of four plant traits (plant size, flowering probability, and number of flowering shoots) differed between countries, while seed mass in situ was similar in both groups (Table 2, Figure 1). Under common garden conditions, plants originating from the introduced populations were larger in size than those originating from the native populations; however, after adjusting for initial size differences, their flowering probability and the number of flowering shoots were smaller (Table 2, Figure 1). Native and introduced populations showed a similar response (if any) to temperature of their origin as indicated by the lack of significant interactions between country and temperature for all four traits considered (Table 2). Temperature of a population's origin explained a significant proportion of the total variation in plant size, with size increasing with increasing mean annual temperature (intercept = 12.873, slope = 0.085 ± 0.209 (*SD*), Table 2). Moreover, seed mass tended to decrease with increasing mean annual temperature (intercept = 3.186, slope = −0.021 ± 0.036 (*SD*), Table 2). Instead, flowering probability and the number of flowering shoots were not associated with temperature of origin (Table 2).

**Table 2:** Results from general and generalized linear mixed models for four traits of the perennial herb *Lupinus polyphyllus*. Population was used as a random factor in all models, and mother plant was further nested within population in the model of seed mass. *df* and ddf denote the degrees of freedom in the numerator and in the denominator, respectively

Response variable	Explanatory variable	χ^2^_df, ddf_	*p‐*value
Seed mass (mg)	Country	1.985_1, 5,113_	.159
	Mean temperature	3.303_1, 5,113_	.069
	Country × Temperature	0.196_1, 5,113_	.658
Size	Country	35.781_1, 361_	<.001
	Mean temperature	4.381_1, 361_	.036
	Country × Temperature	0.794_1, 361_	.373
Flowering prob.	Country	29.365_1, 361_	<.001
	Mean temperature	0.165_1, 361_	.684
	Plant size (early summer)	12.020_1, 361_	<.001
	Country × Temperature	0.447_1, 361_	.504
No. flowering shoots	Country	22.457_1, 163_	<.001
	Mean temperature	0.556_1, 163_	.456
	Plant size (early summer)	7.886_1, 163_	.005
	Country × Temperature	0.205_1, 163_	.650

**FIGURE 2:**
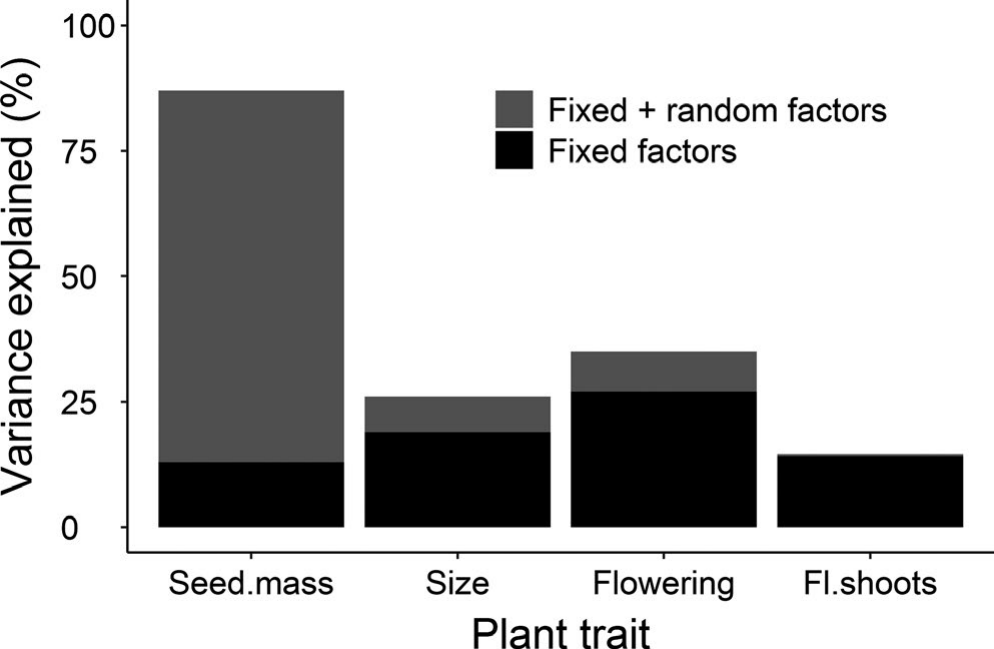
Contributions of the fixed (country and temperature) and random (population) factors to trait variability of the perennial *Lupinus polyphyllus* expressed as marginal and conditional *R* ^2^ values, respectively. For seed mass, mother plant nested within population was also included as a random factor, size was estimated as height × base diameter in cm. Abbreviations are: Size, plant size; Flowering, flowering probability; Fl.shoots, number of flowering shoots

Examination of the marginal and conditional R^2^ values of the statistical models revealed that fixed factors (country and temperature) failed to explain the variation in seed mass (Figure 2). However, inclusion of the random factors (population and mother plant nested within population) greatly improved the explanatory power of the model for this trait (Figure 2), suggesting notable population‐level variation. The opposite was true for variation in the other plant traits, which was primarily explained by the fixed factors, with the addition of the random effect of population improving the explanatory power of the models only a little (Figure 2).

## 4 DISCUSSION

Our results of the first year suggest trait differences in plant size, flowering probability, and the number of flowering shoots (but not in seed mass) between native and introduced populations of *L. polyphyllus*. However, some of these differences (flowering probability and flowering shoot number) were in the opposite direction of our predictions, which calls into question their persistence in the long run as they do not seem to contribute to the species’ invasion success. For both native and introduced populations, temperature of origin contributed to trait variability under common garden conditions, with plant size increasing with increasing mean annual temperature.

When grown under common garden conditions, individuals of *L. polyphyllus* from the introduced populations were larger in size than those originating from the native populations. This finding is in line with the evolution of increased competitive ability (EICA) hypothesis, which proposes that larger size is selected for due to intense intraspecific competition or reduced herbivore pressure in the new range (Blossey & Nötzold, 1995), and could be evidence of postintroduction adaptive evolution. Introduced populations in Finland do indeed form dense stands and experience less herbivory than native populations, which are consumed by multiple different insect herbivores, including seed predators that are currently absent in Finland (Kalske, personal observation). An alternative, and perhaps more likely, explanation for the larger size of *L. polyphyllus* in the introduced populations is pre‐introduction evolution through horticultural plant breeding for ornamental purposes. While the species is diploid in the native range (Sholars & Riggins, 2020), it is polyploid in at least a part of its introduced range (Kubešová et al., 2010; Li et al., 2016). Polyploids are typically more vigorous than diploids (te Beest et al., 2012) and can thus be expected to be larger in size. A third explanation for the larger size of introduced plants is the fact that the common garden was located in their home region, with environmental conditions representing the typical natural conditions of the introduced populations, which might have favoured the performance of these plants. Previous studies have pointed out that plant traits may vary depending on the location of a common garden and that it is preferable to use common gardens in both ranges when comparing plant performance between native and introduced populations (Maron et al., 2004; Moloney et al., 2009). For example, in the perennial herb *Hypericum perforatum*, plants performed best in common gardens that were located at the same latitudes as their source populations (Maron et al., 2004). However, the location of the common garden seems a less likely explanation for the size difference in the present study because the native populations over‐performed the introduced populations in flowering probability and the number of flowering shoots. Finally, the size difference could be due to maternal effects, although this possibility is unlikely because maternal effects tend to be strongest early in life (e.g., Rossiter, 1996) and here plant size was measured after about six months of plant establishment. Regardless of the exact mechanism behind the size difference between the native and introduced populations, large size provides a competitive advantage in resource uptake in the introduced range (van Kleunen et al., 2010), as also explained by Grime's CSR adaptive strategies (e.g., Dalle Fratte et al., 2019).

Despite the fact that plants originating from the introduced populations were larger, we observed that their flowering probability and the number of flowering shoots were lower than those of the native plants under common garden conditions. This observation contradicts previous findings from perennial herbs that individuals tend to be more fecund in the introduced range than in the native range (e.g., reviewed in Parker et al., 2013, but see Elst et al., 2016) or that fecundity does not differ between ranges (Parker et al., 2013; Sun & Roderick, 2019). The inconsistent findings of the present study could be due to the unbalanced study design (i.e., fewer native populations than introduced populations). However, this explanation seems unlikely, given that we sampled a latitudinal gradient of hundreds of kilometers in both ranges. Alternatively, the lower flowering probability and flowering shoot production of introduced plants may not be adaptive, but could have resulted from nonadaptive changes, such as founder effects (Keller & Taylor, 2008). It is also possible that the differences in flowering were due to different photoperiod adaptation. As the native populations represented lower latitudes (Table 1), they were adapted to somewhat shorter days than the introduced populations (daylength = 14.8 hr at latitude 40 and 18.4 hr at latitude 60 in early July). Exposing individuals from lower latitudes to longer days generally hastens flowering of *Lupinus* species (Dracup et al., 1998). Overall, our findings should be interpreted with caution because only two fecundity‐related traits were measured in the common garden, with no direct estimate of seed production. Plants in the field populations of *L. polyphyllus* in Finland can be extremely fecund, producing up to hundreds of seeds per inflorescence (Aniszewski et al., 2001; Ramula, 2014). Therefore, we cannot rule out the possibility that plants in the introduced range produce fewer inflorescences per plant but that these are on average taller and contain more seeds than those in the native range. Moreover, the present study was based on a single growing season and thus provides a snapshot of trait differences early in life. The lower flowering probability in the introduced populations suggests that individuals tend to reach their reproductive stage later than plants in the native populations. It may be that introduced plants have evolved a different life‐history strategy from their native‐range conspecifics, with individuals investing in vegetative growth instead of sexual reproduction early in life. Nevertheless, our observations of the second summer (2020) in the common garden for a subset of individuals (*n* = 22 from the native populations and *n* = 21 from the introduced populations) are similar to those observed in the first summer (2019). In other words, flowering probability and flowering shoot production were lower for plants originating from the introduced populations than for those originating from the native populations also in the second summer (χ^2^ = 9.31, *p* = .002 and χ^2^ = 12.80, *p* < .001 for country in GLMMs, respectively). Further studies based on longer‐term demographic data are required to confirm potential differences in life‐history strategies between native and introduced populations of *L. polyphyllus*.

When temperature of origin was taken into account, seed mass in situ did not differ between the native and introduced populations. This finding is somewhat surprising, given the fact that introduced populations of this species are polyploids that, at least in Finland, have resulted from multiple introductions (Li et al., 2016), which have enabled intraspecific hybridization (admixture). Both polyploidy and multiple introductions with potential hybridization among established populations may promote invasion success and rapid evolution (e.g., Ellstrand & Schierenbeck, 2000; te Beest et al., 2012). Moreover, seed predators, which are present in the native populations of *L. polyphyllus* (Kalske, personal observation), might be expected to exert selective pressure for smaller seed size because plants with lighter seeds can escape predation through better dispersal (Janzen, 1969). On the other hand, seed mass exhibits little variation in some plant species (Harper et al., 1970) and may not necessarily differ in relation to invasion status (e.g., Buckley et al., 2003). For *L. polyphyllus*, seed mass is indeed remarkably similar across introduced populations in different habitat types, although it does vary among individual plants within populations (Sõber & Ramula, 2013). The present study confirms the existence of intraspecific variation in seed mass that is not explained by country or temperature of a population's origin and suggests that seed mass is similar between native and introduced populations. As germination probability increases with increasing seed mass for the study species (Sõber & Ramula, 2013), native and introduced populations might be expected to exhibit a similar germination rate, given their equal seed mass. However, this was not the case in the present study, in which three out of 11 native populations (27%) had poor establishment as compared to one out of 17 introduced populations (6%) under the greenhouse conditions. We do not have an explanation for this difference in seedling establishment; it could be due to either intrinsic (e.g., differences in seed dormancy) or extrinsic (e.g., different photoperiodic adaptation) factors.

Previous comparisons of plant performance between ranges have emphasized the importance of considering climatic differences among populations (Colautti et al., 2009; Rosche et al., 2019). As predicted, we observed that trait variability was partially explained by temperature of a population's origin, with plant size increasing and seed mass tending to decrease with increasing mean annual temperature. Interestingly, the native and introduced populations showed similar clinal variation in relation to this climate variable, which may mean that populations in both ranges are similarly constrained by climate. However, we only considered a single, broad climate variable (mean annual temperature) which may not necessarily reflect local climate conditions. It is thus possible that the native and introduced populations show different clinal variation in relation to other climate variables, such as locally measured precipitation or temperature. In contrast to plant size and seed mass, flowering probability and the number of flowering shoots varied independently of mean annual temperature of a population's origin, suggesting that these traits were less constrained by climate. This result indicates that the species might be able to maintain its reproductive performance under a range of environmental conditions, which supports the hypothesis of the general‐purpose genotype for invasive species (Baker, 1965). However, the plants in the common garden were grown without competitors, and it therefore remains to be tested whether introduced plants are larger and flower less frequently than their conspecific natives also in natural populations. As an example, in the perennial herb *Medicago polymorpha*, introduced individuals were larger than their native conspecifics only in the absence of competition (Getman‐Pickering et al., 2018).

Overall, our hypothesis about higher trait values in the introduced populations of *L. polyphyllus* compared to native populations was only partially supported, because differences in plant performance in relation to invasion status varied depending on the trait in question. Based on the results of the first year, plants from the introduced populations were larger in size, but flowered less frequently and with fewer flowering shoots, than plants from the native populations. Instead, seed mass in situ did not differ between the two groups of populations. Although some of these trait differences (e.g., larger plant size) are likely to be adaptive and contribute to the species’ invasion success, the benefit of increased size in the introduced range might be partially counterbalanced by lower flowering probability and the smaller number of flowering shoots. In addition to invasion status, temperature of a population's origin explained a significant proportion of the variability in plant size under common garden conditions, with plants from both native and introduced populations showing a similar clinal variation in relation to this broad‐scale climate variable. These findings indicate that some traits of the study species may have undergone differentiation between the native and introduced populations. However, the results also raise a question how persistent the trait differences observed in the first year are later in individuals’ life for perennial herbs.

## CONFLICT OF INTEREST

The authors declare no conflict of interest.

## AUTHOR CONTRIBUTION

**Satu Ramula:** Conceptualization (lead); Data curation (lead); Formal analysis (lead); Funding acquisition (lead); Investigation (equal); Visualization (lead); Writing‐original draft (lead); Writing‐review & editing (equal). **Aino Kalske:** Funding acquisition (supporting); Investigation (equal); Writing‐review & editing (equal).

## Data Availability

GenBank accessions, DNA barcoding results, and trait data used for the analyses: Dryad https://doi.org/10.5061/dryad.rjdfn2z8f.
